# Hyaluronate Fragments Reverse Skin Atrophy by a CD44-Dependent Mechanism

**DOI:** 10.1371/journal.pmed.0030493

**Published:** 2006-12-19

**Authors:** Gürkan Kaya, Christian Tran, Olivier Sorg, Raymonde Hotz, Denise Grand, Pierre Carraux, Liliane Didierjean, Ivan Stamenkovic, Jean-Hilaire Saurat

**Affiliations:** 1 Department of Dermatology, University of Geneva, Geneva, Switzerland; 2 Institute of Pathology, University of Lausanne, Lausanne, Switzerland; Medical College of Wisconsin, United States of America

## Abstract

**Background:**

Skin atrophy is a common manifestation of aging and is frequently accompanied by ulceration and delayed wound healing. With an increasingly aging patient population, management of skin atrophy is becoming a major challenge in the clinic, particularly in light of the fact that there are no effective therapeutic options at present.

**Methods and Findings:**

Atrophic skin displays a decreased hyaluronate (HA) content and expression of the major cell-surface hyaluronate receptor, CD44. In an effort to develop a therapeutic strategy for skin atrophy, we addressed the effect of topical administration of defined-size HA fragments (HAF) on skin trophicity. Treatment of primary keratinocyte cultures with intermediate-size HAF (HAFi; 50,000–400,000 Da) but not with small-size HAF (HAFs; <50,000 Da) or large-size HAF (HAFl; >400,000 Da) induced wild-type (wt) but not CD44-deficient (CD44^−/−^) keratinocyte proliferation. Topical application of HAFi caused marked epidermal hyperplasia in wt but not in CD44^−/−^ mice, and significant skin thickening in patients with age- or corticosteroid-related skin atrophy. The effect of HAFi on keratinocyte proliferation was abrogated by antibodies against heparin-binding epidermal growth factor (HB-EGF) and its receptor, erbB1, which form a complex with a particular isoform of CD44 (CD44v3), and by tissue inhibitor of metalloproteinase-3 (TIMP-3).

**Conclusions:**

Our observations provide a novel CD44-dependent mechanism for HA oligosaccharide-induced keratinocyte proliferation and suggest that topical HAFi application may provide an attractive therapeutic option in human skin atrophy.

## Abstract 2

Mouse and human data suggest that topical application of intermediate-size hyaluronate fragments holds therapeutic potential for skin atrophy.

## Editors' Summary

**Background.:**

Time wreaks many changes in the human body but the skin is where one of the first visible signs of aging—wrinkles—occurs. The skin consists of three main layers. The outermost layer is the epidermis. It is the thickness of a sheet of paper and forms a barrier that prevents the body losing water or infectious agents entering it. The cells in the epidermis are mainly keratinocytes. These specialized skin cells are continually produced at the base of the epidermis. From there, they move toward the skin's surface where they are shed. The middle layer is the dermis. It is about ten times thicker than the epidermis and contains the blood vessels that feed the skin, nerves, sebaceous glands, and hair follicles. The final, subcutaneous layer contains sweat glands, some hair follicles, blood vessels and fat. The dermis contains collagen fibers that support the skin and elastin fibers that provide flexibility. Human skin begins to age in early adulthood. By the time a person is 80 years old, their epidermis may be half its original thickness because of decreased keratinocyte proliferation. The dermis also thins, and loss of collagen and elastin fibers means that the skin becomes less elastic. The gradual loss of epidermis and dermis—skin atrophy—is clinically important because aging skin is more fragile and heals slower than young skin and is also prone to ulceration.

**Why Was This Study Done?:**

No one knows why skin atrophy occurs, but it is becoming more common as people live longer, and there is no effective treatment for it. One characteristic of atrophic skin is that, compared to normal skin, it contains less hyaluronate (also called hyaluronan and hyaluronic acid)—a large carbohydrate component of the extracellular matrix, the material that surrounds cells. It also contains less CD44, a cell-surface protein that interacts with hyaluronate. This interaction can stimulate cell proliferation and migration. Given these observations, in this study the researchers have investigated whether treating atrophic skin with fragments of hyaluronate might counteract atrophy.

**What Did the Researchers Do and Find?:**

The researchers isolated keratinocytes from normal mice and from CD44-deficient mice (CD44^−/−^ mice) and treated them with different sized fragments of hyaluronate. Intermediate sized hyaluronate fragments (so-called HAFi) but not large or small fragments increased the proliferation of normal keratinocytes but not CD44^−/−^ keratinocytes. This suggests that proliferation in response to HAFi is CD44-dependent. Similarly, a cream of HAFi applied to the backs of normal mice caused thickening of the epidermal layer but had no effect on CD44^−/−^ mice. Finally, topical application of HAFi for one month caused skin thickening and clinical improvement in six people with skin atrophy but had no effect on normal human skin. The collagen, elastic fiber, and blood vessel content of the dermis also increased in treated patients. By using antibodies to block the function of various proteins, the researchers also discovered that heparin-binding epidermal growth factor (HB-EGF, a protein that stimulates keratinocyte proliferation), erbB1 (a cell-surface protein that binds HB-EGF), and matrix metalloproteinases (proteins that activate HB-EGF) are all required for the stimulation of keratinocyte proliferation by HAFi.

**What Do These Findings Mean?:**

Taken together, these results provide the first indication that application of HAFi to atrophic skin might be useful therapeutically. The absence of any effect on normal human skin is reassuring but puzzling given the thickening seen in normal mouse skin, so this finding needs confirmation before hyaluronate fragments are used clinically. Longer trials in more people are also needed to characterize the clinical effects fully. Finally, the mechanism by which hyaluronate fragments have their effect needs to be studied in more depth. Such studies might reveal other potential therapeutic options for the treatment of skin atrophy.

**Additional Information.:**

Please access these Web sites via the online version of this summary at http://dx.doi.org/10.1371/journal.pmed.0030493.

- MedlinePlus encyclopedia entry on aging changes in skin
- US National Institute on Aging, patient information on skin care and aging
- American Academy of Dermatology, patient information on aging skin
- Information on CD44, the hyaluronan receptor, provided by Glycoforum a source of information on glycobiology
- Wikpedia pages on skin and on hyaluronan (note that Wikipedia is a free online encyclopedia that anyone can edit)

## Introduction

Skin atrophy is a frequent and clinically relevant manifestation of aging, often complicated by ulceration and impaired wound healing. Experimental evidence suggests that defective function of the principal cell-surface hyaluronate (HA) receptor CD44 [1,2], which is associated with impaired HA metabolism, may underlie the pathogenesis of this disorder [3]. HA is the most abundant glycosaminoglycan in cutaneous tissues [4] and is deposited in the extracellular matrix as a high-molecular-weight polymer. Cleavage of the HA polymer during tissue remodeling gives rise to lower-molecular-weight fragments that elicit a variety of CD44-mediated cellular responses, including proliferation, migration, and cytokine synthesis. Increasing evidence suggests that cellular responses elicited by HA depend on the size of the HA oligosaccharides as well as on the responding cell type [5,6]. Thus, HA fragments (HAF), but not native HA polymer or HA disaccharides, could activate Ras and PKCζ in tumor cells in a CD44-dependent fashion [7]; 35,000- to 280,000-Da HA preparations were shown to stimulate CD44-mediated chemokine gene expression in alveolar macrophages [8]; HA oligosaccharides have been reported to induce a toll-like receptor–mediated response in endothelial cells [9]; and only tetra- and hexasaccharide HA fragments have been observed to induce immunophenotypic maturation of human dendritic cells [10].

Cellular responses to HA are primarily mediated by CD44, the most broadly expressed cell-surface HA receptor whose function is regulated at transcriptional, translational, and post-translational levels. Expression levels, variant isoform selection, and glycosylation all play an important role in the ability of CD44 to bind HA [11,12]. Because of its polymeric structure, HA can crosslink several CD44 molecules on the surface of a cell, an effect that is believed to be essential in triggering proliferative, invasive, and promigratory signals [11]. HA-mediated CD44 clustering can have a variety of effects, depending, at least in part, on the CD44 isoform that is implicated in HA binding. Thus, in mouse mammary and uterine epithelia, HA can induce heparan sulfate–bearing variants of CD44 (CD44v3) to recruit a cell-surface complex that includes matrix metalloproteinase 7 (MMP-7), heparin-binding epidermal-like growth factor (HB-EGF) precursor HB-EGF (pro-HB-EGF), and erbB4 [13]. MMP-mediated HB-EGF activation within this complex promotes epithelial cell survival in the lactating mammary gland and postpartum uterus [13]. CD44v3 isoforms are expressed in skin keratinocytes, where HA has been shown to regulate keratinocyte proliferation [3]. CD44 in turn appears to play an important role in maintaining HA homeostasis in skin [3], and its levels appear to correlate with skin trophicity. Thus, decreased epidermal CD44 expression is associated with the pathogenesis of an atrophic skin disorder known as lichen sclerosus et atrophicus [14]. Conversely, epidermal hyperplasia induced by topical administration of retinoids in mouse skin is accompanied by increased CD44 and HA synthase (HAS) expression with a concomitant increase in HA deposition [15]. To elucidate the mechanism whereby HA might induce keratinocyte proliferation, and to determine the response of the epidermis to its application in vivo, we examined the effect of HAF of varying size on in vitro–cultured mouse keratinocyte growth, and skin trophicity of DBA/1 wild-type (wt) and CD44^−/−^ mice SKH1 hairless mice, as well as normal and atrophic human skin. Our observations indicate that HAF of 50,000–400,000 Da, defined as intermediate-size HAF (HAFi), induce keratinocyte proliferation and skin hyperplasia by a CD44-dependent mechanism that requires proteolytic activation of HB-EGF and engagement of erbB1.

## Methods

### Preparation of HAFi

HA from rooster comb (IAL) for clinical application was provided by Transbussan (http://www.transbussan.com). Three types of HA from IAL were used: (1) HAFl: high-molecular-weight HA (IAL); (2) HAFi: fragmentation products of HA of medium size (50,000–400,000 Da) was prepared using a Braun sonifier (http://www.bbraunusa.com) for 30 min at 400 W on ice. The fragments were then separated on a Sephacryl S-400 HR 4 × 80 cm size exclusion gel filtration column (Pharmacia Diagnostics, http://www.phadia.com). The column was calibrated using Pullulan standards (Showa-Denko, http://www.showadenko.com) from 20,000 to 800,000 Da. Fractions (100) of 10 ml each were collected from the column with a Frac 100 fraction collector (Pharmacia, http://www.pharmacia.com) at a flow rate of 1 ml/min. The HA concentration of each sample was determined by a colorimetric dosage according to a BCA-reducing sugar assay, and an elution profile for the fragments was obtained. The fractions corresponding to the 50,000–400,000 Da were pooled, dialyzed against distilled water, and lyophilized. (3) HAFs: small fragmentation products of HA (1,000–50,000 Da) were generated by enzymatic digestion of high-molecular-weight HA with bovine testis hyaluronidase (Sigma, http://www.sigmaaldrich.com) for 1 h at 37 °C in 0.1 M sodium acetate buffer, 0.15 M NaCl, and 1mM EDTA (pH 5.0), and lyophilized. The fragments were separated by 15% polyacrylamide gel (TBS) electrophoresis and visualized with an alcian blue and silver staining. The HAF of different sizes were prepared in the form of cream by the same vehicle.

### Treatment of Mice

Groups of five adult (>3 mo-old) SKH1 hairless, DBA/1 (The Jackson Laboratory, http://www.jax.org), or CD44-deficient (CD44^−/−^) mice [13] were used. HAF (0.2%) or vehicle cream samples of 0.5 g were applied twice daily for 3 d to the dorsal skin of SKH1 hairless, DBA/1, or CD44^−/−^ mice. Biotinylated 0.2% HAF (Carbomer, http://www.carbomer.com) in Liposol (Sigene, http://www.sigene.com) was applied twice daily for 3 d on the back skin of SKH1 hairless mice. TPA (Sigma; 0.005% solution in acetone) was applied twice daily for 3 d to the dorsal skin of DBA/1 or CD44^−/−^ mice. Animals were killed 2 h after the last application. All experiments were approved by the Ethical Commission on Animal Experimentation of the University of Geneva and the Cantonal Veterinary Office of Geneva.

### Treatment of Healthy Participants and Patients

Seven healthy young adults (seven males) between 19 and 32 y (mean age, 25.5 y), ten healthy women in menopause without hormone replacement therapy between 55 and 65 y (mean age, 60 y), three patients with advanced age-related skin atrophy (two females, one male) between 60 and 88 y (mean age, 76 y) and three patients with skin atrophy due to prolonged use of oral corticosteroids for rheumatoid arthritis (three females) between 74 and 86 y (mean age, 81 y) were included in this study after obtaining informed consent. Clinical studies were conducted with the authorization and according to the guidelines of Ethical Commission on Human Research of the University Hospital of Geneva. HAFi (1%) or vehicle cream samples of 0.5 g were applied twice daily for 1 mo on the posterior side of the right or left arm, respectively.

### Histology

Dorsal skin samples were fixed in 10% phosphate-buffered formaldehyde, embedded in paraffin, and processed for histological analysis. Sections were cut at 5 μm, mounted onto slides, and stained with hematoxylin-eosin, Sirius red, and van Gieson elastin according to standard procedures.

### Staining of Skin Sections for CD44, CD44v3, HA-Binding Protein, Ki-67, Biotinylated HAF, Vimentin, Filaggrin, Loricrin, K14, and CD31

Paraffin-embedded sections (5 μm) were mounted onto slides, dewaxed in xylene, rehydrated in a graded ethanol series, and prepared for immunoperoxidase staining according to standard procedures. Primary antibodies included anti-CD44v3 (1:100; Bender MedSystems, http://www.bendermedsystems.com), anti–Ki-67 (1:20; Dako, http://www.dako.com), anti-filaggrin (1:750; Abcam, http://www.abcam.com), anti-loricrin (1:500; Abcam); anti-K14 (1:100; Novocastra, http://www.novocastra.co.uk), and anti-CD31 (1:100; Dako). After staining with the primary reagent for 1 h at room temperature, sections were washed, incubated with biotinylated affinity-purified secondary antibody or with biotinylated anti-CD44 (2.5 μg/ml; PharMingen, http://www.bdbiosciences.com) or HA-binding protein (HABP, 25 ng/ml; Seikagaku Kogyo, Japan, http://www.seikagaku.co.jp) for 30 min at room temperature, washed, and treated with avidin-biotin-peroxidase for 30 min at room temperature. The sections were then washed with buffer and incubated in 0.05% DAB (3,3′-diaminobenzidine; Sigma) and 0.03% H_2_O_2_ in phosphate buffer at room temperature. Cryostat sections (5 μm) of skin samples of mice treated with biotinylated HAF were frozen in liquid nitrogen, mounted onto slides, air-dried, and incubated with anti-vimentin antibody (1:20; Santa Cruz Biotechnology, http://www.scbt.com) for 1 h at room temperature. The sections were then washed and incubated with biotinylated immunoglobulins for anti-vimentin antibody and then streptavidin-FITC (1:500; Invitrogen, http://www.invitrogen.com) and streptavidin rhodamine (1:500; Pierce Biotechnology, http://www.piercenet.com). All sections were examined under a Zeiss axiophot microscope (http://www.zeiss.com) using appropriate filters.

### Hyaluronidase Digestion

Hyaluronidase treatment of tissues was performed by incubating the tissue sections with 1.5 μg/ml bovine testicular hyaluronidase (Sigma) in phosphate-buffered saline (PBS) for 5 h at 37 °C. The sections were then stained either with colloidal iron or with HABP as described above. The hyaluronidase digestion experiments also included negative controls incubated under otherwise similar conditions but lacking the enzyme. An HA-secreting mesothelioma section was used as a positive control.

### Epidermal and Cutaneous Thickness Measurements

Epidermal thickness of mice was measured by a graded ocular and multiplied by ten to correct the scale. Cutaneous thickness measurements of the healthy participants and patients were performed using a skin ultrasound system (Episcan; Longport, http://www.longportinc.com).

### Cell Proliferation

Epidermal and dermal Ki-67^+^ cells were counted in ten fields per section at 40× magnification; the average value was calculated.

### Western Blot Analysis

Frozen mouse epidermis or dermis and human biopsy samples were incubated in extraction buffer containing 20 mM Tris-HCl (pH 7.5), 100 mM NaCl, 10 mM EDTA, 1% SDS, 10% glycerol, and protease inhibitor cocktail (complete; Boehringer, http://www.boehringer-ingelheim.com), minced, polytron-homogenized, and sonicated on ice. Cell culture extracts were treated with the same buffer. Homogenates were spun in a microcentrifuge for 20 min, and the soluble fraction was extracted and subjected to western blot analysis with appropriate antibodies.

Samples were loaded in nonreducing SDS sample buffer, subjected to electrophoresis, and transblotted onto 0.45 μm pore–size nitrocellulose membrane. Antibodies used for Western blot analysis were anti-CD44 standard (Bender MedSystems); anti-CD44v3 (Bender MedSystems); anti–MMP-7 (G-20; Santa Cruz Biotechnology); anti–pro-HB-EGF (M-18; Santa Cruz Biotechnology); anti–HB-EGF neutralizing antibody (R&D Systems, http://www.rndsystems.com); and anti-erbB1 and anti-erbB4 (Santa Cruz Biotechnology).

### Immunoprecipitation

Lysates were incubated overnight at 4 °C with anti-CD44 antibodies and protein A+G agarose beads (Pierce Biotechnology) in the presence of BSA and rat IgG. Elution of antigen–antibody complex was performed in nonreducing SDS sample buffer by heating 5 min at 95 °C. After centrifugation for 3 min at 15,000*g*, supernatant was loaded on a 10% polyacrylamide gel.

### Quantification of CD44, HA, and erbB1

Quantification of CD44, HA, and erbB1 in the skin samples by an enzyme-linked binding protein assay was performed using the sCD44 ELISA Kit (Bender MedSystems), the Corgenix Hyaluronic Acid Quantitative Test Kit (Endotell, http://www.endotell.ch), and the erbB1 ELISA Kit (Sigma), respectively, according to the manufacturers' instructions.

### In Vitro Keratinocyte Proliferation Assay

Epidermal keratinocytes were isolated and cultured in 96-well plates (Becton Dickinson, http://www.bd.com) as described previously [16]. On day 2 of culture, HAFs, HAFi, or HAFl (100 μg/ml), monoclonal anti-human amphiregulin (AR) neutralizing antibody (100 ng/ml), monoclonal anti-human erbB1 neutralizing antibody (isotype IgG1; 100 ng/ml), mouse recombinant tissue inhibitor of metalloproteinase 3 (TIMP-3; 100 ng/ml), or human HB-EGF (5 ng/ml; R&D Systems), TPA (1 ng/ml; Sigma), and human EGF (50 ng/ml; Sigma), in the presence or absence of anti–HB-EGF neutralizing antibody (10 ng/ml; R&D Systems), was added to the cultures. Mouse IgG1 was used as a control of anti-erbB1. Human fibroblasts and human umbilical vein endothelial cells (HUVECs) were cultured in 96-well plates. Culture medium contained DMEM, 10% FCS, 1% antibiotics for the fibroblasts; and M199, 10% FCS, 1% antibiotics, glutamine, 1% heparin, 1% ECGS (endothelial cell growth supplement; Upstate, http://www.upstate.com), 1% hydrocortisone, and 1% vitamin C for the HUVECs. On day 2 of culture, bFGF (1 ng/ml), VEGF (20 ng/ml), or HAFi (100 μg/ml) was added to the cultures. On day 5 of culture, supernatants alone or with HAFi (100 μg/ml) were added to the keratinocyte cultures (1:4 ratio). Later (48 h), 0.037 MBq of [^3^H]thymidine (Amersham, http://www.amersham.com) was added to each well. Isotope incorporation was evaluated 24 h later in a Beckman LS 1801 (http://www.beckmancoulter.com). β counter (Beckman Coulter, Fullerton, California). All experiments were done in triplicate and repeated 5 times, and the results were expressed as the mean of incorporated counts per minute for each condition tested.

## Results

### HAFi Induce CD44-Dependent Keratinocyte Proliferation and Skin Hyperplasia

**Figure 1:**
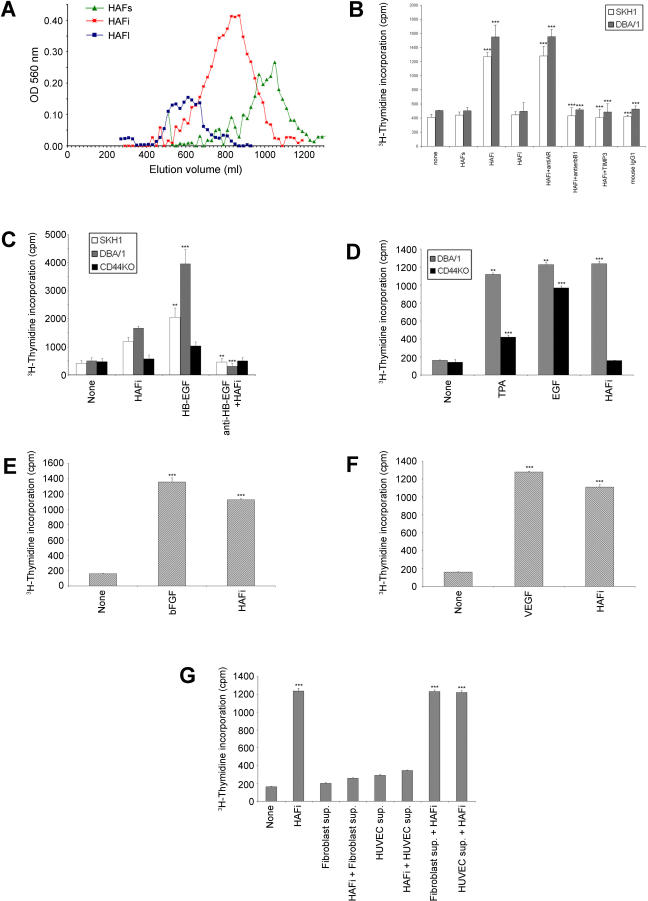
The Effect of HAF In Vitro (A) Elution profile of HAF. HAFs, HAFi, or HAFl were run on a Sephacryl S-400 column after sonification and enzymatic digestion. (B) HAFi, but not HAFs or HAFl, induces in vitro mouse keratinocyte proliferation that is inhibited by anti-erbB1 antibody and TIMP-3. Keratinocytes from SKH1 and DBA/1 wt^−^ mice were cultured in 96-well plates. On day 5 of culture, HAFs, HAFi, or HAFl (100 μg/ml), monoclonal anti-human AR neutralizing antibody (100 ng/ml), monoclonal anti-human erbB1 neutralizing antibody (isotype IgG1; 100 ng/ml), or mouse recombinant TIMP-3 (100 ng/ml) was added to the cultures. Mouse IgG1 was used as a control for anti-erbB1. Later (48 h), 0.037 MBq of [^3^H]-thymidine was added to each well. All experiments were done in triplicate and repeated five times. The results are presented as the mean incorporated counts per min ± standard error of the mean (SEM) of three wells per group. ***p < 0.001 (HAFi and HAFi + anti-AR versus none; HAFi + anti-erbB1, HAFi + TIMP3, and mouse IgG1 versus HAFi; Student's t-test). (C) In vitro proliferative response of mouse keratinocytes to HAFi is CD44 and HB-EGF dependent. Keratinocyte proliferation in response to HAFi in vitro. Keratinocytes from SKH1, DBA/l wt, and DBA/l CD44^−/−^ mice were cultured in 96-well plates. On day 5 of culture, HAFi (100 μg/ml), human HB-EGF (50 ng/ml), or mouse anti-human HB-EGF–neutralizing antibody (100 ng/ml) was added to the cultures. Later (48 h), 0.037 MBq of [^3^H]-thymidine was added to each well. All experiments were done in triplicate and repeated five times. The results are presented as the mean incorporated counts per min ± SEM of three wells per group. **p < 0.01; ***p < 0.001 (HB-EGF versus none; anti–HB-EGF + HAFi versus HAFi; Student's t-test). (D) Keratinocyte proliferation in response to TPA and EGF in vitro. Keratinocytes from DBA/l wt and DBA/l CD44^−/−^ mice were cultured in 96-well plates. On day 5 of culture, TPA (1 ng/ml), EGF (50 ng/ml), or HAFi (100 μg/ml) was added to the cultures. Later (48 h), 0.037 MBq of [^3^H]-thymidine was added to each well. All experiments were done in triplicate and repeated five times. The results are presented as the mean incorporated counts per min ± SEM of three wells per group. **p < 0.01 versus none; ***p < 0.001 versus none (Student's t-test). (E) Fibroblast proliferation in response to bFGF in vitro. Human fibroblasts were cultured in 96-well plates. On day 2 of culture, bFGF (1 ng/ml) or HAFi (100 μg/ml) was added to the cultures. Later (48 h), 0.037 MBq of [^3^H]-thymidine was added to each well. All experiments were done in triplicate and repeated five times. The results are presented as the mean incorporated counts per minute ± SEM of three wells per group. ***p < 0.001 versus none (Student's t-test). (F) Vascular endothelial cell proliferation in response to VEGF in vitro. HUVECs were cultured in 96-well plates. On day 2 of culture, VEGF (20 ng/ml) or HAFi (100 μg/ml) was added to the cultures. Later (48 h), 0.037 MBq of [^3^H]-thymidine was added to each well. All experiments were done in triplicate and repeated five times. The results are presented as the mean incorporated counts per min ± SEM of three wells per group. ***p < 0.001 versus none (Student's t-test). (G) Keratinocyte proliferation in response to HAFi-treated fibroblast or HUVEC supernatants in vitro. Keratinocytes from DBA/l wt mice, fibroblasts, or HUVECs were cultured in 96-well plates. On day 2 of culture, HAFi (100 μg/ml) was added to the fibroblast or HUVEC cultures. On day 5 of culture, supernatants alone or supplemented with HAFi (100 μg/ml) were added to the keratinocyte cultures (1:4 ratio). Later (48 h), 0.037 MBq of [^3^H]-thymidine was added to each well. All experiments were done in triplicate and repeated five times. The results are presented as the mean incorporated counts per min ± SEM of three wells per group. ***p < 0.001 versus none (Student's t-test).

Cultured keratinocytes from the dorsal skin of wt and CD44^−/−^ DBA/1 mice were incubated with HAF generated from high-molecular-weight HA by sonication, enzymatic digestion, and size exclusion gel filtration (Figure 1A). Exposure to HAFi but not to HAFl or HAFs resulted in increased keratinocyte proliferation (Figure 1B). Consistent with the notion that the proliferative response to HA is CD44 mediated, HAFi failed to induce proliferation in CD44^−/−^ keratinocytes (Figure 1C).

To verify that the inability of HAFi to induce proliferation of CD44^−/−^ keratinocytes was indeed due to the lack of CD44 and not to a more general proliferation defect of these cells, we assessed the response of CD44^−/−^ keratinocytes to phorbol ester and EGF stimulation. CD44^−/−^ keratinocytes were observed to mount a proliferative response to both mitogens, albeit to a lesser degree than wt cells (Figure 1D). It would therefore seem reasonable to attribute the complete lack of keratinocyte response to HAFi to the absence of the HA receptor CD44.

The stimulatory effect of HAFi was not limited to cultured keratinocytes. Incubation of primary human fibroblasts from a healthy donor and HUVECs with HAFi at concentrations that induced keratinocyte proliferation resulted in increased proliferation of both cell types (Figure 1E and 1F).

**Figure 2:**
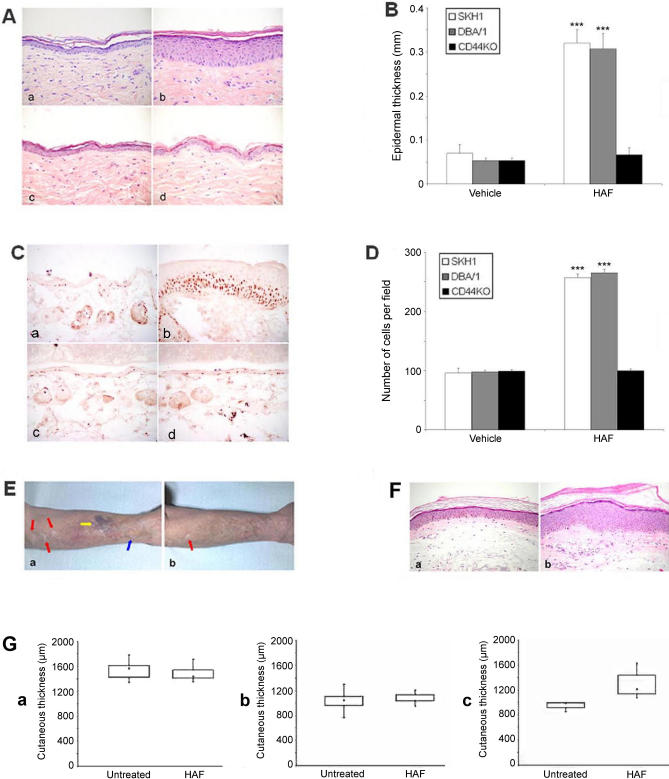
The Effect of HAF on Mouse and Human Skin (A) HAFi-induced hyperplasia of mouse skin is CD44 dependent. Histological sections of vehicle-treated (a and c) or HAFi-treated (b and d) DBA/1 (a and b) or CD44^−/−^ (c and d) dorsal mouse skin. Note the epidermal hyperplasia in DBA/1 but not in CD44^−/−^ mice. (B) Epidermal thickness in vehicle- or HAFi-treated SKH1, DBA/1, and CD44^−/−^ mouse back skin measured with an ocular micrometer. Ten measurements were performed per mouse, and the average value was calculated. The results are presented as the mean epidermal thickness ± SEM of six animals per group. ***p < 0.001 versus vehicle (Student's t-test). (C) In vivo proliferative response of mouse epidermis to HAFi is CD44 dependent. Ki67 staining of vehicle-treated (a and c) or HAFi-treated (b and d) DBA/1 (a and b) or CD44^−/−^ (c and d) dorsal mouse skin. (D) Dermal cellularity in vehicle- or HAFi-treated SKH1, DBA/1, and CD44^−/−^ mouse back skin. Samples were counted at 40× magnification. Ten counts were made per mouse, and the average value was calculated. The results are presented as the mean number of cells ± SEM of six animals per group. ***p < 0.001 versus vehicle (Student's t-test). (E) HAFi corrects age- and corticosteroid-related atrophy in human skin. Atrophic human forearm skin 1 mo after topical treatment with vehicle (a) or 1% HAFi (b). Note the decrease of wrinkles, hemorrhage (yellow arrow), and pseudoscars (red arrows), the visibility of superficial vessels (blue arrow), and the smoothening of the skin with after HAFi treatment. (F) HAFi corrects age- and corticosteroid-related atrophy in human skin. Histology of atrophic human forearm skin 1 mo after topical treatment with vehicle (a) or 1% HAFi (b). Note the significant epidermal hyperplasia after HAFi treatment. (G) HAFi results in skin hyperplasia in atrophic but not normal human skin. Skin thickness in HAFi-treated young (a), nonlesional aged (b), or atrophic aged (c) human skin measured by echography. The results are presented as boxplots with median values (triangles). Young untreated versus nonlesional aged untreated, *p* < 0.001; young untreated versus atrophic aged untreated, *p* = 0.001; atrophic aged untreated versus atrophic aged treated, *p* < 0.01 (nonparametric Mann-Whitney U test).

To determine whether HA fragments may induce keratinocyte proliferation in vivo, the effect of repeated local administration of HAFi to the dorsal skin of wt and CD44^−/−^ DBA/l mice was assessed. To control for possible strain specific effects, SKH1 hairless mice were also subjected to local HAFi treatment. Daily topical application of a solution of 0.2% HAFi for 3 d resulted in significant epidermal hyperplasia (Figure 2A and 2B) that reflected increased cell proliferation, as demonstrated by increased numbers of epidermal and dermal Ki-67^+^ cells, in all six wt but in none of the six CD44^−/−^ mice tested (Figure 2C and 2D). Consistent with the behavior of CD44^−/−^ keratinocytes in vitro, the skin of CD44^−/−^ mice displayed moderate hyperplasia in response to local phorbol ester application (Figure S1A and S1B), supporting the notion that the complete lack of hyperplasia in response to HAFi could be attributed to the absence of CD44 itself.

The potential effect of HAFi on keratinocyte differentiation in vivo was assessed by staining of HAFi- and vehicle-treated skin sections with antibodies against differentiation markers, including keratin-14, filaggrin, and loricrin. All three differentiation markers were found to display increased expression in hyperplastic HAFi-treated skin that was proportional to the degree of hyperplasia (Figure S2A). Thus, keratin-14 expression was observed throughout the epidermis, whereas filaggrin and loricrin expression was confined to the granular layer, suggesting that HAFi application had no obvious effect on keratinocyte differentiation in vivo.

The effect of HAFi on the composition of the dermis was assessed by addressing changes in collagen expression and vascularization. Staining with Sirius red revealed an increase in collagen content of the superficial dermis (Figure S2B [parts a and b]). Elastic fiber increase in the dermis was revealed by van Gieson elastin staining (Figure S2B [parts c and d]), and an increase in blood vessel content was observed with anti-CD31 antibody (Figure S2B [parts e and f]), reflecting the effect of HAFi on fibroblasts and HUVECs observed in vitro.

Finally, to determine whether HAFi-mediated stimulation of fibroblasts and endothelial cells might indirectly participate in keratinocyte proliferation, keratinocytes derived from wt mice were incubated with 1:4 diluted conditioned culture media from 72-h HAFi- and vehicle-treated fibroblasts and HUVECs. Neither HAFi-treated fibroblasts nor HUVEC culture supernatants displayed any significant effect on keratinocyte proliferation in vitro (Figure 1G). However, supplementation of the 72-h HAFi-treated endothelial cell and fibroblast supernatants with 100 μg/ml of fresh HAFi induced keratinocyte proliferation to a degree that was similar to that when HAFi were directly added to keratinocyte cultures (Figure 1G), rendering unlikely the possibility that nutrient depletion of the supernatants inhibited proliferation. It would seem conceivable that the HAFi used to stimulate endothelial cells and fibroblasts had been internalized and/or degraded by 72 h of culture, providing a plausible explanation for the lack of keratinocyte stimulation by the 72-h conditioned culture media. Taken together, these observations suggest that HAFi-induced epidermal hyperplasia in vivo reflected a direct effect of HAFi on keratinocyte proliferation, independent of fibroblast and endothelial cell stimuli.

### Topical Application of HAFi Restores Atrophic Skin Lesions in Humans

To assess the effect of HAFi administration to human skin, six patients with atrophic skin lesions and 17 control participants, including seven healthy men (age, 29–32 y; mean age, 25.5 y) and ten healthy postmenopausal women who had not received hormone replacement therapy (age, 55–65 y; mean age, 60 y) were subjected to daily topical application to the forearm of a 1% preparation of HAFi for 1 mo. Following termination of the treatment, none of the control participants revealed a measurable increase in skin thickness, signs of inflammation, or scaling (Figure 2G [part a]). By contrast, all six patients with skin atrophy that was either age-related (three patients, aged 60–88 y; mean age, 76 y) or associated with corticosteroid therapy for rheumatoid arthritis (three patients, aged 74–86 y; mean age, 81 y) responded to topical HAFi application by developing marked skin thickening (Figure 2E and 2F, and Figure 2G [part b]) at the end of the treatment period. Most notably, pseudoscars (representing the healing stage of hyperextended skin; Figure 2E, red arrows), hemorrhage (Figure 2E, yellow arrow), and visible superficial vessels (Figure 2E, blue arrow), all of which are associated with skin atrophy, disappeared in response to HAFi-mediated restoration of skin trophicity (Figure 2E and 2F). Contrary to HAFi, the same concentration of HAFl and HAFs applied for the same duration to the six patients with skin atrophy had no effect on skin thickness (unpublished data).

### HAFi Applied to the Skin Induce Expression of CD44 and Enzymes Implicated in HA Metabolism

**Figure 3:**
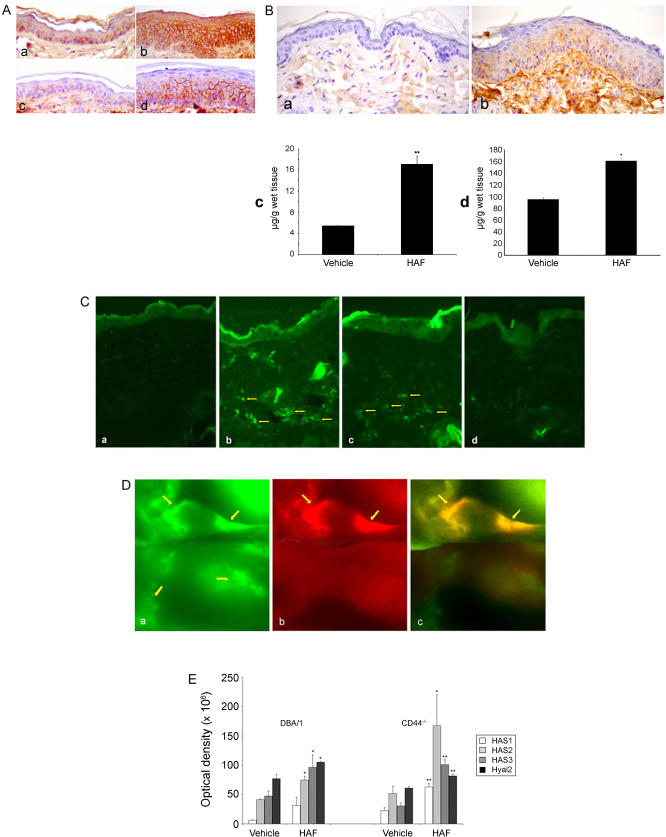
The Effect of HAF on CD44, HA, and HA-Polymerizing and -Degrading Enzymes, and Localization of HAF in Mouse Skin (A) HAFi increases CD44 expression in mouse skin. Immunostaining of sections of vehicle-treated (a and c) or HAFi-treated (b and d) DBA/1 mouse dorsal skin with anti-CD44 (a and b) or anti-CD44v3 (c and d) antibodies. Note the hyperplasia and increase in both CD44 and CD44v3 expression in the epidermis. (B) HAFi increases the epidermal and dermal HA content in mouse skin. HABP staining of sections of vehicle-treated (a) or HAFi-treated (b) DBA/1 mouse dorsal skin showing elevated amounts of HA in the dermis. Epidermal (c) and dermal (d) HA content of HAFi-treated skin of SKH1 hairless mice were quantified by an enzyme-linked binding protein assay. The results are presented as the mean HA concentration ± SEM of six animals per group. **p < 0.01 versus vehicle; *p < 0.05 versus vehicle (Student's t-test). (C) HAFs and HAFi penetrate mouse skin. Streptavidin-FITC staining of sections of vehicle (a), biotinylated HAFs-treated (b), HAFi-treated (c), or HAFl-treated (d) SKH1 hairless mouse back skin. Note the presence of biotin in HAFs-treated, and to a lesser extent, in HAFi-treated skin (arrows). (D) Topically applied HAFi is located both intra- and extracellularly. Sections of HAFi-treated dorsal SKH1 hairless mouse skin were stained with streptavidin-FITC (a) or anti-vimentin antibody, biotinylated secondary antibody, and streptavidin-rhodamine (b). Topically applied HAFi (a; green fluorescence, top arrows) shows colocalization (c; yellow fluorescence, arrows) with vimentin (b; red fluorescence, arrows) and outside the cells in the extracellular matrix (a; lower arrows). (E) HAFi increases the expression of HASs and Hyal2 in mouse skin. Northern blot analysis of HAS1, HAS2, HAS3, and Hyal2 RNA expression in vehicle- or HAFi-treated DBA/1 wt or CD44^−/−^ mouse dorsal skin. The hybridization signals were quantitated by scanning the autoradiograms with a laser densitometer. The results are presented as the mean optical density ± SEM of three animals per group. *p < 0.05 versus vehicle; **p < 0.01 versus vehicle (Student's t-test).

To begin to address the putative mechanism of HAFi-induced skin hyperplasia, we assessed changes in CD44 expression and HA synthesis in response to local HA application. Topical HAFi application, but not HAFl or HAFs (unpublished data) application, resulted in increased CD44 expression at the RNA and protein levels throughout the epidermis (Figure 3A [parts a and b] and unpublished data). HAFi also selectively increased CD44v3 isoform expression in basal and suprabasal keratinocytes, with the exception of the granular layer (Figure 3A [parts c and d]).

The superficial dermis of HAFi-treated DBA/1 mouse dorsal skin sections displayed strong reactivity with biotinylated HABP, an established probe of tissue HA (Figure 3B), which was abrogated by hyaluronidase pretreatment of the tissue sections (unpublished data). An enzyme-linked binding protein assay confirmed the presence of increased amounts of HA in the epidermis and dermis (Figure 3B [parts c and d] and unpublished data) of HAFi-treated DBA/l and SKH1 hairless mice.

To address the fate of locally administered HAF to the skin, size-fractionated HA was biotinylated, and its localization was traced with fluorescein-labeled streptavidin. Biotinylated HAFs-treated SKHI hairless mice and, to a lesser extent, HAFi- but not HAFl-treated SKH1 hairless mice displayed biotin deposits in the superficial dermis after 3 d of topical application (Figure 3C). The observed biotin deposits were removed by hyaluronidase treatment of the skin sections (unpublished data), consistent with the notion that both HAFs and HAFi can penetrate the epidermis. To further assess HAFi localization in tissues, double staining using biotinylated HAFi and fluorescein-labeled streptavidin as well as anti-vimentin antibody was performed. Colocalization of HAFi and vimentin suggested internalization of HAFi by mesenchymal dermal cells whose morphology was consistent with that of fibroblasts (Figure 3D and unpublished data). HAFi were also observed in vimentin-negative acellular areas, consistent with the extracellular matrix.

To determine whether the absorbed HAF might affect local HA synthesis and degradation, we assessed the expression of HA-polymerizing enzymes HAS1, HAS2, and HAS3 and the major tissue HA-degrading enzyme, hyaluronidase 2 (Hyal2), in HAFi-treated skin of DBA/1 and CD44^−/−^ mice (Figure 3E). Northern blot analysis showed an increase in HAS1, HAS2, HAS3, and Hyal2 transcripts in both wt and CD44^−/−^ skin, suggesting that HAFi can induce both HA-producing and -degrading enzymes in a CD44-independent manner.

### HAFi-Mediated Keratinocyte Proliferation is HB-EGF and EGFR Dependent

**Figure 4:**
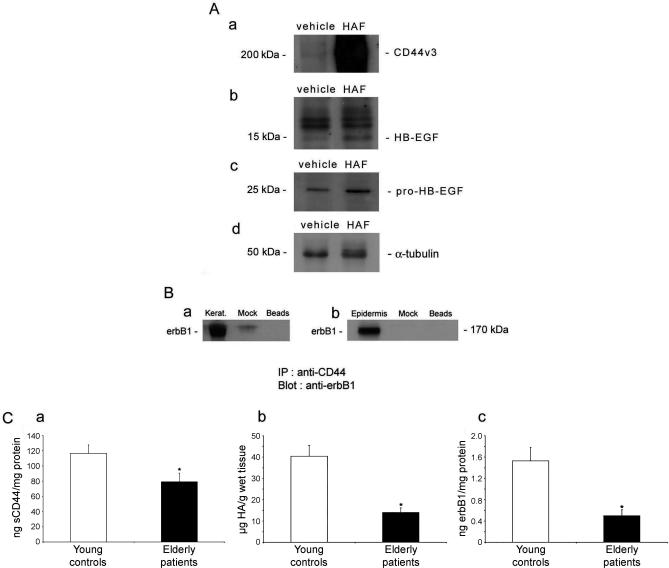
The Effect of HAF on CD44v3, pro-HB-EGF, and HB-EGF in Mouse Skin; CD44 and erbB1 Association In Vitro and In Vivo; CD44, HA, and erbB1 Levels in Young and Elderly Skin (A) HAFi induces expression of CD44v3, pro-HB-EGF, and active HB-EGF in mouse skin. Western blot analysis on the protein extracts of vehicle- or HAFi-treated SKH1 hairless mice for CD44v3 (∼200 kDa [a]), active HB-EGF (∼15 kDa [b]), pro-HB-EGF (∼25 kDa [c]), and loading control α-tubulin (50 kDa [d]). (B) CD44 associates with erbB1 in keratinocytes in vitro and in vivo. Western blot analysis of anti-CD44 antibody immunoprecipitates of protein extracts of cultured keratinocytes (a) or epidermis of DBA/1 mouse skin immunoblotted with anti-erbB1. Mock, isotype-matched rat IgG; beads, protein A+G agarose beads treated with anti-CD44 antibody. (C) CD44, HA and erbB1 levels are diminished in atrophic human skin. CD44 (a), HA (b), and erbB1 (c) expression in forearm skin biopsy specimens of young adults (control) and elderly patients with skin atrophy. The results are presented as the mean CD44, HA, or erbB1 concentration ± SEM of three subjects per group. *p < 0.05 versus young controls (Student's t-test).

Because HAFi-induced CD44v3 expression (Figure 3A [parts c and d]), we addressed the expression of molecules reported to be recruited by CD44v3, particularly HB-EGF and its receptors [13], in HAFi-treated mouse skin. HAFi-treated DBA/l and SKH1 hairless epidermis displayed a robust induction of CD44v3 and pro-HB-EGF (22–28 kDa) expression as well as a mild induction of erbB1 expression (Figures 4A and S3, and unpublished data). Importantly, cleaved (14–19 kDa) HB-EGF species, corresponding to the active form, appeared in lysates of HAFi-treated epidermis (Figure 4A). As erbB4 is not expressed in mouse skin [17], erbB1 fulfils the role of the principal HB-EGF receptor. To assess the physical association between CD44 and erbB1, we performed coimmunoprecipitation experiments. Consistent with observations showing interactions between CD44 and erbB4 [13], as well as erbB2 and erbB3 [18], Western blot analysis using anti-erbB1 antibody of anti-CD44 antibody immunoprecipitates from lysates of cultured keratinocytes and epidermis of DBA/1 mouse skin revealed that erbB1 coimmunoprecipitates with CD44 (Figure 4B). It is noteworthy that the skin of patients with atrophy displayed decreased CD44 and erbB1 expression as well as reduced HA synthesis (Figure 4C).

Based on the observations that HAFi treatment enhanced CD44v3, erbB1, and HB-EGF expression and induced HB-EGF activation, we addressed the possibility that these molecules may form part of the functional machinery implicated in the regenerative response of keratinocytes to HAFi. The proliferative response of wt DBA/1 mouse keratinocytes to HAFi was abrogated by anti-human erbB1 neutralizing antibody (Figure 1B). Recombinant HB-EGF stimulated robust proliferation of wt DBA/1 keratinocytes but induced only mild proliferation of CD44-deficient counterparts, whereas AR, which does not bind CD44v3 [19], had no effect. Neutralizing anti–HB-EGF antibody inhibited the HAFi-induced keratinocyte proliferation in wt keratinocytes (Figure 1C), whereas an isotype-matched unrelated antibody failed to do so. Because MMPs and the related ADAM family proteases are implicated in proteolytic activation of pro-HB-EGF, we tested the effect of the MMP and ADAM (a disintegrin and metalloproteinase) inhibitor TIMP-3 on HAFi-induced keratinocyte proliferation. Consistent with the notion that MMPs [13] and ADAMs [20] activate HB-EGF, recombinant TIMP-3 abrogated HAFi-induced keratinocyte proliferation (Figure 1B).

## Discussion

Our observations provide evidence for the first time to our knowledge that topically applied HAF ranging from 50,000 to 400,000 Da penetrate the epidermis and induce keratinocyte proliferation that translates into the thickening of mouse and human skin. Penetration of topically applied HA of 250,000–400,000 Da into mouse and human dermis was recently demonstrated [21]. Here we show that both HAFs and HAFi penetrate mouse skin in vivo, but that only the HAFi induce cellular proliferation within the epidermal and dermal compartments.

At least two types of effects occurred as a result of topical HAFi application: CD44-independent penetration of skin and induction of HAS and Hyal2, and CD44-dependent keratinocyte proliferation. Both fibroblasts and endothelial cells also proliferated in response to HAFi in vitro, providing a possible explanation for the increased dermal collagen deposition and angiogenesis, respectively, observed in vivo. However, the contribution of fibroblast activity and angiogenesis to epidermal hyperplasia was most likely of minor importance, given that conditioned culture media of HAFi-stimulated fibroblasts and endothelial cells failed to augment keratinocyte proliferation. Importantly, an increase in human skin thickness in response to HAFi was observed only in patients with skin atrophy. Although the reasons of the absence of such a response in healthy participants can only be speculative at present, it is possible that physiological HA production saturates tissue CD44 binding capacity in the steady state, such that additional exogenous HA fragments fail to induce a significant CD44-dependent response. Whatever the precise mechanism, the absence of skin hyperplasia in healthy participants in response to HAFi suggests that topical HAFi administration does not present the risk of inducing undesirable local side effects.

Penetration of HA fragments into the dermis may occur via hair follicles, which provide a well-recognized route for macromolecular skin penetration [22] and could explain the dermal localization of HAFs and HAFi. However, the increase in dermal HA content of HAFi- and HAFs-treated hairless SKH1 mouse skin, which contains incompletely developed and partially functional follicles [22], leaves open the possibility for the participation of an additional putative mechanism of size-limited HAF penetration, including possibly passive absorption. HAFi was observed to induce HAS expression consistent with the possibility that increased local HA synthesis may contribute to the increased HA content. The mechanism whereby HAFi might induce local HAS and hyaluronidase expression is unknown. Keratinocytes and fibroblasts provide the principal source of HA in the epidermis and dermis, respectively [23], and control local HA metabolism. CD44 is believed to play a major role in the uptake of HA by receptor-mediated endocytosis [24] in both cell types. Following internalization, HA undergoes intracellular degradation that is possibly mediated by endosomal/lysosomal hyaluronidases [25]. HAF released by keratinocytes traverse the basement membrane to the dermis, where they are cleared via lymphatic vessels [26]. It would appear that mechanisms independent of CD44 might sense changes in local HA concentration, or that HAFi stimulate receptors other than CD44 to induce HAS and hyaluronidase expression. Increased tissue HA concentration typically occurs in the context of development, injury, and tumor growth, and is believed to actively participate in the process of tissue remodeling. It is conceivable that an increase in tissue HA, whether of endogenous or exogenous origin, is interpreted by local fibroblasts to reflect a remodeling process, triggering additional HA synthesis and degradation.

Proliferation in response to HAFi is a CD44-dependent event. Our present observations provide evidence that in addition to CD44, HB-EGF, erbB1, and MMPs/ADAMs are required for HA-dependent in vitro keratinocyte proliferation. The absence of a proliferative response of CD44^−/−^ keratinocytes to HB-EGF is consistent with the notion that HB-EGF interaction with its receptors requires presentation by heparan sulfate side chains of CD44v3-containing isoforms [19]. Similar to other members of the EGF family, pro-HB-EGF is expressed as an integral membrane protein of 22–28 kDa [27], which, following stimulation of cells with mitogens, undergoes MMP-mediated release of mature soluble 14–19 kDa HB-EGF [28]. Accordingly, topical application of HAFi oligosaccharide resulted in a significant increase in pro-HB-EGF and HB-EGF, while inhibition of MMP activity by TIMP-3 abrogated the corresponding proliferative response.

**Figure 5:**
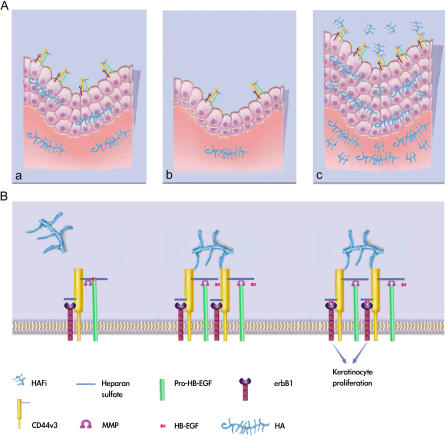
Hypothetical Assembly of the CD44v3/HB-EGF/erbB1 Complex and the Mechanism of HAFi-Induced Keratinocyte Proliferation (A) CD44, HA, and HB-EGF in normal skin (a), atrophic skin (b), and following treatment of atrophic skin treated with HAFi (c). (B) Schematic representation of the hypothetical assembly of the putative complex: HAFi mediates CD44v3 and heparan sulfate–bound pro-HB-EGF aggregation, resulting in the recruitment and activation of an MMP/ADAM. Pro-HB-EGF is cleaved by the MMP/ADAM, and the resulting active moiety binds and activates erbB1, generating proliferation signals.

The observation that anti-erbB1– and anti–HB-EGF–blocking antibodies had the same abrogating effect as the absence of CD44 on in vitro keratinocyte proliferation in response to HAFi supports the notion that erbB1 signaling, triggered by HB-EGF, may play a key role in HAFi-induced proliferation. Similar to its role in uterine and mammary epithelia, HA-induced CD44v3 aggregates may recruit a functional cell-surface complex in keratinocytes composed of pro-HB-EGF, erbB1, which replaces erbB4, and an MMP or ADAM [13,20]. MMP/ADAM-mediated proteolytic cleavage of HB-EGF and CD44-mediated presentation of mature HB-EGF to erbB1 may then induce proliferation (Figure 5).

Our observations have defined 50,000–400,000 Da HAF as reagents capable of inducing a proliferative response in mouse and human skin. Clinically, topical HAFi application resulted in epidermal hyperplasia with restoration to normal thickness of atrophic human skin as early as 1 mo after initiation of treatment. This effect was accompanied by significant clinical improvement, suggesting that HAFi may provide the basis for the development of novel therapeutic strategies for skin diseases characterized by atrophy.

## Supporting Information

Figure S1 The Effect of TPA on CD44^−/−^ Mouse Skin (A) TPA induces a reduced skin hyperplasia in CD44^−/−^ mice. Histological sections of acetone-treated (parts a and c) or TPA-treated (parts b and d) DBA/1 (parts a and b) or CD44^−/−^ (parts c and d) dorsal mouse skin. Note the decreased epidermal hyperplasia in CD44^−/−^ mice.
(B) Epidermal thickness in acetone- or TPA-treated DBA/1 and CD44^−/−^ mouse back skin measured with an ocular micrometer. Ten measurements were performed per mouse, and the average value was calculated. The results are presented as the mean epidermal thickness ± SEM of six animals per group. ***p < 0.001 versus acetone (Student's t-test).
(293 KB JPG)

Figure S2 The Effect of HAFi on Mouse Epidermal Differentiation and Atrophic Human Dermis (A) HAFi has no effect on epidermal differentiation in mouse skin. Immunostaining of sections of vehicle-treated (parts a, c, and e) or HAFi-treated (parts b, d, and f) DBA/1 mouse dorsal skin with anti-K14 (parts a and b), anti-filaggrin (parts c and d), or anti-loricrin (parts e and f) antibody. Note the lack of increase in staining intensity despite the increased number of stained cells in HAFi-treated skin.
(B) HAFi increases the collagen, elastin and vascular content in atrophic human skin. Histology of atrophic human forearm skin 1 month after topical treatment with vehicle (part a) or 1% HAFi (part b), stained with Sirius red (parts a and b), van Gieson elastin (parts c and d), or anti-CD31 antibody (parts e and f). Note the increase in dermal collagen, elastic fibers, and vessels after HAFi treatment.
(882 KB JPG)

Figure S3 HAFi Induces Expression of CD44v3 in Mouse Skin Western blot analysis on the protein extracts of vehicle- or HAFi-treated SKH1 hairless mice for CD44v3.
(454 KB JPG)

## Abbreviations

ADAM;: a disintegrin and metalloproteinase
AR: amphiregulin
EGF: epidermal-like growth factor
HA: hyaluronate
HABP: HA-binding protein
HAF: HA fragment(s)
HAFi: intermediate-size HAF
HAFl: large-size HAF
HAFs: small-size HAF
HAS: HA synthase
HB-EGF: heparin-binding EGF
HUVEC: human umbilical vein endothelial cell
MMP-7: matrix metalloproteinase 7
pro-HB-EGF: HB-EGF precursor
SEM: standard error of the mean
TIMP-3: tissue inhibitor of metalloproteinase-3
wt: wild-type
